# Changes in conceptions in women younger than 18 years and the circumstances of young mothers in England in 2000–12: an observational study

**DOI:** 10.1016/S0140-6736(16)30449-4

**Published:** 2016-08-06

**Authors:** Kaye Wellings, Melissa J Palmer, Rebecca S Geary, Lorna J Gibson, Andrew Copas, Jessica Datta, Anna Glasier, Rachel H Scott, Catherine H Mercer, Bob Erens, Wendy Macdowall, Rebecca S French, Kyle Jones, Anne M Johnson, Clare Tanton, Paul Wilkinson

**Affiliations:** a Centre for Sexual and Reproductive Health Research, London School of Hygiene & Tropical Medicine, London, UK; b Department of Health Services Research and Policy, London School of Hygiene & Tropical Medicine, London, UK; c Faculty of Public Health and Policy, London School of Hygiene & Tropical Medicine, London, UK; d Research Department of Infection and Population Health, University College London, London, UK

## Abstract

**Background:**

In 2000, a 10-year Teenage Pregnancy Strategy was launched in England to reduce conceptions in women younger than 18 years and social exclusion in young parents. We used routinely collected data and data from Britain's National Surveys of Sexual Attitudes and Lifestyles (Natsal) to examine progress towards these goals.

**Methods:**

In this observational study, we used random-effects meta-regression to analyse the change in conception rates from 1994–98 to 2009–13 by top-tier local authorities in England, in relation to Teenage Pregnancy Strategy-related expenditure per head, socioeconomic deprivation, and region. Data from similar probability sample surveys: Natsal-1 (1990–91), Natsal-2 (1999–2001), and Natsal-3 (2010–12) were used to assess the prevalence of risk factors and their association with conception in women younger than 18 years in women aged 18–24 years; and the prevalence of participation in education, work, and training in young mothers.

**Findings:**

Conception rates in women younger than 18 years declined steadily from their peak in 1996–98 and more rapidly from 2007 onwards. More deprived areas and those receiving greater Teenage Pregnancy Strategy-related investment had higher rates of conception in 1994–98 and had greater declines to 2009–13. Regression analyses assessing the association between Teenage Pregnancy Strategy funding and decline in conception rates in women younger than 18 years showed an estimated reduction in the conception rate of 11·4 conceptions (95% CI 9·6–13·2; p<0·0001) per 1000 women aged 15–17 years for every £100 Teenage Pregnancy Strategy spend per head and a reduction of 8·2 conceptions (5·8–10·5; p<0·0001) after adjustment for socioeconomic deprivation and region. The association between conception in women younger than 18 years and lower socioeconomic status weakened slightly between Natsal-2 and Natsal-3. The prevalence of participation in education, work, or training among young women with a child conceived before age 18 years was low, but the odds of them doing so doubled between Natsal-2 and Natsal-3 (odds ratio 1·99, 95% CI 0·99–4·00).

**Interpretation:**

A sustained, multifaceted policy intervention involving health and education agencies, alongside other social and educational changes, has probably contributed to a substantial and accelerating decline in conceptions in women younger than 18 years in England since the late 1990s.

**Funding:**

Medical Research Council, Wellcome Trust, Economic and Social Research Council, and Department of Health.

## Introduction

At the end of the 20th century, concern for high rates of teenage conception in the UK compared with other western European countries, together with the strong association between early parenthood and deprivation, provided the impetus to public health efforts to prevent teenage pregnancy. In 1999, the UK Government launched a 10-year, nationwide Teenage Pregnancy Strategy^1^ in England with the dual aims of achieving a 50% reduction in conception rates in women younger than 18 years by 2010, and mitigating social exclusion in teenage parents by increasing their participation in education, employment, or training.^2^ A strong rationale for the strategy was the desire to halt the cycle of deprivation resulting from the increment of disadvantage conferred by early pregnancy additional to that experienced before conception.^3^, ^4^, ^5^, ^6^

The teenage pregnancy strategy gave rise to a multicomponent programme comprising a national media campaign; improvements to sex education and young people's sexual health services; support for young parents to increase participation in education, training, and employment; and joint action to ensure national and local coordination across statutory and voluntary agencies.^7^ Financial resources were allocated according to teenage pregnancy rates in each top-tier local authority area. Because of the concentration of high rates of teenage pregnancy in poorer areas of the country, financial resources were disproportionately invested in the more deprived areas of England. Scotland, Wales, and Northern Ireland decided their initiatives independently.

**Research in context**

**Evidence before this study**

We searched PubMed for studies published between Jan 1, 2000, and March 31, 2016, with no language restrictions using the search terms “teenage pregnancy”, “teenage conception”, “trends”, and “public health policy” to identify studies describing trends in teenage pregnancy and policy-related factors associated with change, in the UK and elsewhere. We examined the titles and abstracts identified by the search, excluding those that were not relevant, and reviewed the full text of the remaining articles to assess appropriateness for inclusion.

Few studies were able to measure direct indicators of intervention-related action, and none combined general population survey data and routinely collected statistics. Most studies were from the USA and the UK, settings in which the prevalence of teenage pregnancy is high compared with other high-income countries. Existing studies have near universally reported strong associations between higher rates of teenage pregnancy and lower socioeconomic level. They have, however, differed in their interpretation of whether the decline in the prevalence of teenage pregnancy in the 21st century can be attributed to broader societal changes, such as increased educational attainment, or to public health interventions such as increased use of reliable contraception.

**Added value of this study**

Our study aimed to assess progress towards the goals of the Teenage Pregnancy Strategy mounted in England to reduce conception rates in women younger than 18 years, combining analyses of data from a population-based survey and from routinely collected national statistics. The scale of the decline in conception rates in women younger than 18 years, and its association with intervention-related investment and with both demographic and behavioural factors, suggest a combined influence of both public health intervention and secular trends on the decline in conceptions in women younger than 18 years in England.

**Implications of all the available evidence**

The trend towards postponement of key life events such as completion of education, leaving home, starting employment, and settling with a partner is now near universal. These factors seem to have contributed to a global trend towards fewer early pregnancies. Coincidental with this, focused and sustained efforts to lower the prevalence of early pregnancy by raising awareness, changing social norms, and increasing access to education and reliable contraception seem to have accelerated the trend towards fewer teenage pregnancies.

Conception rates in women younger than 18 years fell by 51% between 1998 and 2014 in England. Of interest is the extent to which the decline seems to be related to the Teenage Pregnancy Strategy and other interventions; whether the decline has been seen equally in the most and least disadvantaged women; and what factors remain associated with conception in women younger than 18 years. In this study, we combined analyses of routinely available area-level data for conceptions, deprivation, and policy-related expenditure, and individual-level data from three decennial waves of the National Survey of Sexual Attitudes and Lifestyles (Natsal) to describe change in outcomes relating to key goals of the English Teenage Pregnancy Strategy—ie, conception rates in women younger than 18 years and the prevalence of participation in education, work, and training in women with a child conceived before the age of 18 years.

## Methods

### Study design and participants

In this observational study, we analysed routine data for births and abortions for top-tier local authorities (top-tier local authorities consist of London boroughs, metropolitan borough councils, and unitary authorities, and are a level of local government with responsibilities including education and social services) in England from 1994 to 2013 and individual-level data from three waves of the National Survey of Sexual Attitudes and Lifestyles (wave 1: 1990–91 [Natsal-1], wave 2: 1999–2001 [Natsal-2], and wave 3: 2010–12 [Natsal-3]). We obtained data by calendar year for the resident population and births and abortions by age of mother from the Office for National Statistics for each of the top-tier local authorities in England. Because of their small resident populations, two authorities (City of London and the Scilly Isles) were combined with more populous neighbouring authorities (Hackney and Cornwall, respectively), resulting in a total of 148 local authorities for analysis.

We also obtained data from the Department for Communities and Local Government at the top-tier local authority level for the 2004 Index of Multiple Deprivation, a score of socioeconomic deprivation based on a weighted average of 38 separate indicators across seven distinct domains (employment, income, health and disability, education skills and training, barriers to housing and other services, crime, and living environment).^8^

As an indicator of the extent of Teenage Pregnancy Strategy-related local activity, we obtained from the Department for Children, Schools and Families the Teenage Pregnancy Strategy annual Local Implementation Grant awarded to each top-tier local authority for the financial year(s) 1999–2000 and 2010–11, the amount reflecting the challenge in terms of conception rates in women younger than 18 years and the size of the population. From these data we calculated the total investment per head by dividing the amount by the 2001 Office of National Statistics estimate of resident women aged 13–17 years. We obtained ethics approval for Natsal-2 from University College Hospital, North Thames Multicentre, and all local research ethics committees in Britain, and for Natsal-3 from the Oxford Research Ethics Committee A.

### Procedures

The Natsals are probability sample surveys of British residents of whom 18 876 aged 16–59 years were interviewed between May, 1990, and November, 1991 (Natsal-1); 11 161 aged 16–44 years were interviewed between May, 1999, and February, 2001 (Natsal-2); and 15 162 aged 16–74 years between September, 2010, and August, 2012 (Natsal-3). Participants resident in London were oversampled in Natsal-2, and those aged 16–34 years were oversampled in Natsal-3. The unadjusted response rate in Natsal-1 was 64·7%, and the cooperation rate (of eligible addresses contacted) was 71·5%. The unadjusted response rate for Natsal-2 was 63·1% and the adjusted rate, taking account of oversampling in London, was 65·4%. The response rate for Natsal-3 was 57·7% and the cooperation rate was 65·8%.

In Natsal-1, paper questionnaires were self-completed and interviewer administered. In Natsal-2 and Natsal-3, participants were interviewed with a combination of computer-assisted personal and computer-assisted self-interviews. Experimental comparison of pencil and paper and computer-assisted interviewing revealed no important differences in responses.^9^ For all three surveys, after correcting for unequal selection probabilities, a non-response post-stratification weight was applied to ensure comparability with census data in terms of age, sex, and region. Further details of the methods and response calculations are described elsewhere.^10^, ^11^

Natsal-1 and Natsal-2 asked about the number and timing of all abortions and livebirths; Natsal-3 asked for timing and outcome of each pregnancy. We calculated conceptions that occurred before age 18 years among 18–24-year-old women by summing births that occurred before age 18 years 9 months and abortions occurring before age 18 years, excluding miscarriages and stillbirths, for consistency with Office of National Statistics procedures. We assessed change in the prevalence of participation in any of education, work, or training in young mothers.

Variables used in the analyses presented in this paper were selected on the basis of pre-existing evidence of their association with early conception.^3^, ^4^, ^5^, ^6^ They include age at first heterosexual intercourse; use of a reliable method of contraception at first sex; consensuality in terms of being equally or more willing than the partner at time of first sex; autonomy of decision making—ie, not influenced by peer pressure or use of drugs or alcohol; and retrospective views on the timing of first sex—ie, whether it occurred sooner or later than ideal or at the right time. We also included the main source of information about sexual matters at the start of sexual activity, adequacy of that information, and ease of communication with parents about sex. Demographic measures included age at interview, family structure (whether the participant lived with both parents until age 16 years [Natsal-2] or age 14 years [Natsal-3]), educational attainment, and area-level deprivation measured with the Index of Multiple Deprivation.^12^ Full postcode data for use in estimating the Index of Multiple Deprivation were not available for Natsal-1.

### Statistical analysis

Analyses using routinely collected data included tabulation and graphical representation of trends in conception rates of women younger than 18 years by quartiles of per head total Local Implementation Grant investment and quartiles of area-level deprivation. Random-effects meta-regression analyses of the change in conception rates of women younger than 18 years between 1994–98 (the pre-intervention baseline) and 2009–13 (the most recently available Office of National Statistics data) were based on aggregate top-tier local authority level data. Because of variability in how each top-tier local authority spent their grant, a random-effects meta-regression analysis was used. Absolute and percentage changes between these timepoints were analysed as outcomes separately and data from each top-tier local authority were weighted by the inverse of the variance of the outcome estimate. The explanatory factors considered were per head Teenage Pregnancy Strategy investment, region, and deprivation (2004 Index of Multiple Deprivation scores). For the two continuous covariates (Teenage Pregnancy Strategy investment and Index of Multiple Deprivation score), the linearity of their association with the outcome variable (change in conception rate in women younger than 18 years) was assessed by also including quadratic terms in the regression model.

Analyses using Natsal data included logistic regression to examine factors associated with conception in women younger than 18 years separately in 1999–2001 and 2010–12 in women aged 18–24 years, with and without adjustment for the effect of all variables except age at first intercourse. Logistic regression with interaction terms was also used to assess whether associations between factors and conception in women younger than 18 years had changed between 1999–2001 and 2010–12. Analyses using Natsal data also included bivariate tabulations and logistic regression to examine the prevalence in 1990–91, 1999–2001, and 2010–12 of participation in work, education, or training in women aged 18–24 years, according to whether or not they had a child conceived before age 18 years. Logistic regression with interaction terms was used to assess whether the association between early motherhood and participation changed over time. All analyses were done with Stata 12.1 (version 13; StataCorp LP, College Station, Texas), accounting for stratification, clustering, and weighting of the Natsal data.

### Role of the funding source

The funders had no role in study design, data collection, data analysis, data interpretation, or writing of the report. The corresponding author had full access to all the data in the study and had final responsibility for the decision to submit for publication.

## Results

**Figure 1:**
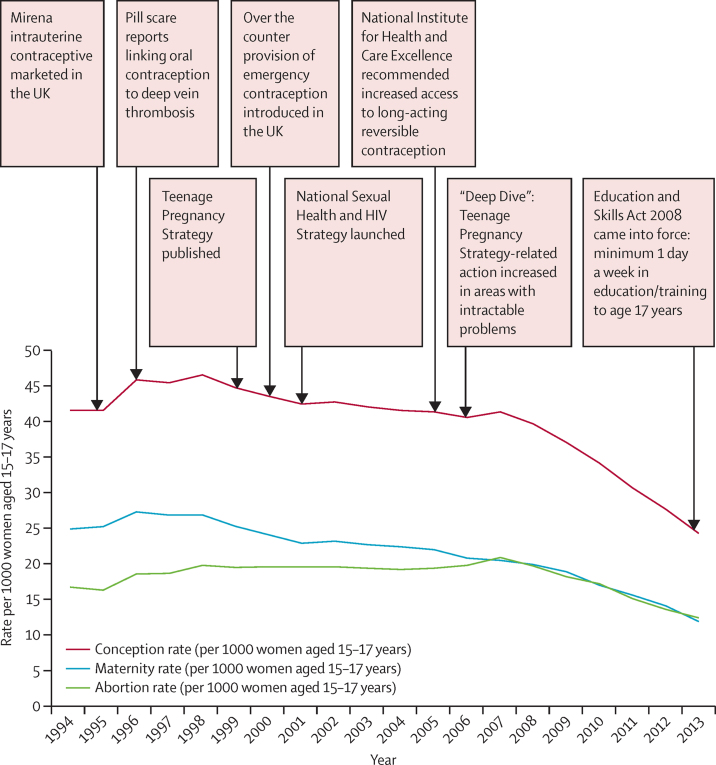
Conception, maternity, and abortion rates and events of possible relevance to trends in women younger than 18 years between 1994 and 2013 (per 1000 women aged 15–17 years)

Routinely collected national data for conceptions in women younger than 18 years show a steady decline from their peak in 1998, with an apparent acceleration in that decline from 2007 onwards (figure 1). The trend was initially driven by the decline in maternities, until 2007 when the plot lines for abortions and births converged.

**Figure 2:**
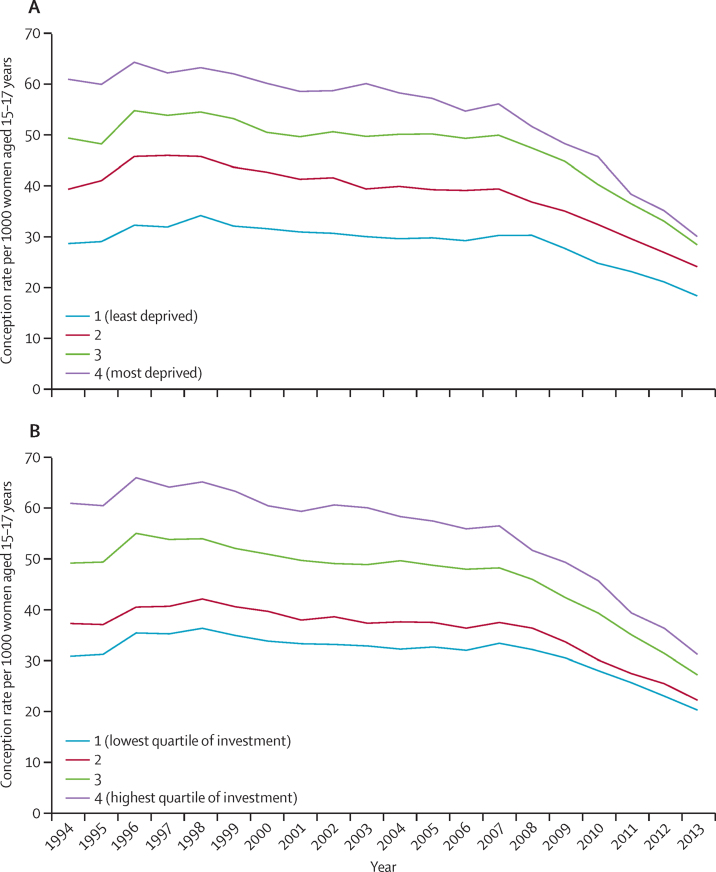
Conception in women younger than 18 years by quartile between 1994 and 2013 by Index of Multiple Deprivation (A) and Local Implementation Grant investment for Teenage Pregnancy Strategy (B)

The annual rates of conception in women younger than 18 years by quartile of area-related deprivation between 1994 and 2013 declined across all deprivation levels but this decline was larger in the most deprived areas (figure 2). Between 1998 and 2013, for example, conception rate declined by 16 conceptions per 1000 women aged 15–17 years in the least deprived areas compared with 33 conceptions per 1000 women aged 15–17 years living in the most deprived areas. This differential decline has resulted in a partial convergence in conception rates in women younger than 18 years across the quartiles of deprivation.

The annual data for the same period by quartile of strategy-related expenditure at local level—ie, per head total Local Implementation Grant investment—showed partial gradual convergence in conception rates in women younger than 18 years across Local Implementation Grant quartiles, with the greatest decline in areas receiving the highest Local Implementation Grant award (figure 2). Between 1998 and 2013, areas receiving the highest Local Implementation Grant award had a decline of 34 conceptions per 1000 women aged 15–17 years, whereas in areas receiving the lowest level, the decline was 16 conceptions per 1000 women aged 15–17 years.

Regression analyses assessing the association between Teenage Pregnancy Strategy funding and decline in conception rates in women younger than 18 years showed an estimated reduction in the conception rate of 11·4 (95% CI 9·6–13·2; p<0·0001) per 1000 women aged 15–17 years for every £100 Teenage Pregnancy Strategy spend per head (table 1). After adjustment for socioeconomic deprivation and region, this trend retained statistical significance, with a reduction of 8·2 conceptions (5·8–10·5; p<0·0001) per 1000 women aged 15–17 years per £100 Teenage Pregnancy Strategy spend per head. This trend was also reflected when modelling the percentage change in conception rates in women younger than 18 years as the outcome; for every £100 Teenage Pregnancy Strategy spend per head, a 6·2% (95% CI 2·3–10·2) reduction in the conception rate in women younger than 18 years was reported after adjusting for region and deprivation. Testing of quadratic terms indicated that associations with Teenage Pregnancy Strategy spend per head (and Index of Multiple Deprivation score) were approximately linear over the range of values considered for both absolute and percentage change, although the two models are not strictly compatible. The value of *I*^2^ for both meta-regression analyses was almost 100% because the outcome estimates from the top-tier local authorities are all very precise as they are based on rates from tens of thousands of person-years, and hence the variability seen is interpreted as variability in the underlying change across top-tier local authorities.

The prevalence and odds ratios (ORs) of conception in women younger than 18 years by selected characteristics of women aged 18–24 years in 1999–2001 (Natsal-2) and 2010–12 (Natsal-3) are shown (table 2). Comparisons between the two surveys must be cautiously made because of minor but possibly important differences in question formulation. However, with this proviso and within the limits of precision of the estimates for the 18–24-year-old age group, the results for the two survey waves are broadly similar with regard to the prevalence of behaviour likely to be proximally and causally associated with conception. The proportion with more than minimum academic qualifications was, however, appreciably higher in 2010–12 (Natsal-3) compared with 1999–2001 (Natsal-2), as was the proportion reporting school lessons as their main source of sex education. A more modest increase between the two timepoints in the proportion reporting use of reliable contraception at first intercourse was noted.

Lower socioeconomic status, lower educational attainment, earlier onset of sexual activity, receiving sex education from sources other than school and, more weakly, negative opinion about the timing of first intercourse were associated with higher conception in women younger than 18 years in both surveys. Interaction testing revealed little clear evidence of change in the association with conception in women younger than 18 years between surveys for any of the factors considered (all p values were greater than 0·05 and are not shown). However, the association between living in an area in the highest quintile of deprivation and conception in women younger than 18 years was somewhat weaker in Natsal-3 than in Natsal-2, (Natsal-2 1999–2001: adjusted OR [AOR] 5·18 [95% CI 1·91–14·05]); Natsal-3 2010–12: AOR 2·89 [1·25–6·67]). A similar finding was seen for educational level. Having minimum or no academic qualifications was strongly associated with conception in both surveys, but the AOR was somewhat lower in Natsal-3 than in Natsal-2 (Natsal-2 1999–2001: AOR 5·61 [2·97–10·62]; Natsal-3 2010–12: AOR 3·61 [2·29–5·67]). The weaker association between use of reliable contraception at first intercourse and conception in women younger than 18 years was further attenuated between Natsal-2 and Natsal-3.

Results from Natsal-1, Natsal-2, and Natsal-3 relating to the participation of women aged 18–24 years according to whether or not they had conceived a child before age 18 years are shown (table 3). Estimates should be treated with caution because of the comparatively small survey subsamples in the 18–24-year-old age group. The results for all three surveys show the proportion of women in education, work, or training at the time of interview was higher by a considerable order of magnitude in those who did not conceive a child before 18 years than in those who did (table 3). However, although the likelihood of participation of women who were not young mothers was unchanged across the three surveys (1999–2001 *vs* 1990–91: OR 1·21 [95% CI 0·93–1·58]; 2010–12 *vs* 1999–2001: 0·86 [0·67–1·11]), in young mothers it remained constant between Natsal-1 and Natsal-2, but doubled between Natsal-2 and Natsal-3 (1999–2001 *vs* 1990–91: 1·12 [0·56–2·25]; 2010–12 *vs* 1999–2001: 1·99 [0·99–4·00]). Consequently, the association between young motherhood and participation weakened substantially between Natsal-2 and Natsal-3.

## Discussion

We report a marked decline in conceptions in women younger than 18 years, which was greater in areas of greater deprivation and also in areas of higher Teenage Pregnancy Strategy investment. The steep deprivation gradient previously associated with conception in women younger than 18 years has been partly attenuated. Similarly, the association between conception before age 18 years and lower educational level remains significant but has weakened over the period. Young people increasingly learn about sexual matters mainly from school lessons, and the association between conception before age 18 years and receiving sex education from other sources remains strong. The prevalence of participation in education, work, and training in women with a child conceived before age 18 years, although low, increased between 1999–2001 and 2010–12, a trend not seen in other, same-aged women.

Reductions in rates of teenage pregnancy—ie, those occurring before age 20 years—have been seen in other high-income countries,^13^ indicating a broader secular trend. Fewer comparative data exist specifically on pregnancies in women younger than 18 years, and hardly any include abortion data. However, data for the birth rate in women younger than 18 years in the European Union between 2004 and 2014 show a decline from 13·6 to 6·8 per 1000 women aged 15–17 years in the UK compared with the average of 7·7 to 6·0 per 1000 women for all 28 countries (Office of National Statistics, unpublished). Moreover, the weakening of the association between conception rates in women younger than 18 years and deprivation in our study reverses the previous trend. In the 1980s and 1990s, teenage pregnancy rates increased in women living in the most deprived areas but remained unchanged or decreased in those in more affluent areas.^14^, ^15^ The increase in economic participation of teenage mothers seen in our data after 2000, is also a reversal of a previous trend. In 2005, we reported data from Natsal-1 and Natsal-2,^16^ which showed a widening gap at the end of the 20th century in the life chances and material prosperity of women who became mothers at an early age and those who did not. The recent improvement has occurred despite the less favourable economic climate in the post-2008 recession period, when the disparity between rich and poor has increased.

The decline in teenage pregnancy rates has been differentially attributed to distal factors such as increased educational attainment and to more proximal factors such as improved use of contraception. Researchers finding a strong association between falling teenage pregnancy rates and increasing use of long-acting reversible contraception in England since the late 1990s have underlined the contribution of increased access to reliable contraception to the decline.^17^ Observers in the USA have reached similar conclusions^18^ supported by intervention studies examining the effect of providing highly effective contraception free of charge.^19^ By contrast, researchers using ecological analyses of routinely collected area-level data in England to examine the association between educational attainment, contraceptive use, and trends in conception in women younger than 18 years concluded that the larger association with education indicates that this is the key driver.^20^ Interpreting the associations between educational level, effective contraception, and early pregnancy is difficult because of the likelihood of reverse causality. Low educational attainment is both a cause and consequence of teenage pregnancy. The effect of use of highly reliable contraception in reducing early conception rates might be masked in research by the tendency for women who experience early pregnancy to subsequently use more reliable methods. Our findings suggest that contraceptive use and educational attainment each wield an independent influence on the likelihood of conception in women younger than 18 years. Because Natsal asks about contraception in the last year and ever, we are unable to measure use at the time of conception, and so reliable method use at first intercourse has been used as a proxy indicator in these analyses. However, unpublished Natsal data show a two-fold increase in use of long-acting reversible contraception methods by sexually active 16–17 year olds between Natsal-2 and Natsal-3.

Both increases in educational attainment and in use of highly effective contraception are likely to have contributed to the falling teenage pregnancy rate. Educational aspirations provide the motivation and contraception the means by which to avert early pregnancy. Both are clearly policy related. Other studies have shown that multiple interventions (combining educational and behavioural interventions) lower the rate of teenage pregnancy^21^ and highlight the importance of education in planning policies with this as their aim.^22^ This was reflected in the decision by the English Government to locate the unit responsible for the Teenage Pregnancy Strategy jointly in two government ministries, the Department of Education and the Department of Health, and to make joint working between education and health agencies a key component of the strategy.

A strength of this study lies in the combination of individual-level and area-level data, and in our capacity to show independent associations between conception rates in women younger than 18 years and possibly influential variables. However, slight changes in question wording between the surveys might have influenced responses relating to pregnancy in Natsal-2 and Natsal-3. In particular, we should note that abortions were slightly under-reported in Natsal-3 compared with the official UK figure in 2011.^23^

Compared with experimental approaches, observational studies have limitations in assessing the relative effect of policy-related intervention and secular trends on health outcomes. Specifically in this case, Teenage Pregnancy Strategy funding was determined by the pre-intervention conception rate. This creates challenges in disentangling the effect of Teenage Pregnancy Strategy funding from the effects of the pre-intervention rate on the subsequent conception rate, including the potential for regression to the mean. Our finding of an association between the decline in conception rates in women younger than 18 years and Teenage Pregnancy Strategy resourcing is suggestive that government-linked efforts have contributed towards lowering conception rates in women younger than 18 years but should not be seen as conclusive.

Individual-level-data have also enabled us to show, where analyses of area-related data have not,^24^ the progress that has been made in reducing the previously strong link between deprivation and early conception. The comparatively greater decline in conception rate in women younger than 18 years in the most deprived areas is worthy of note. Although the potential for decline might have been greater where baseline conception rates were higher, these were often areas in which complex and multiple social and health problems competed for public health efforts and resources.

Progress has been made towards halting the cycle of poverty and income inequality long associated with early pregnancy, and in improving the life chances of young mothers. Despite this success, England's teenage pregnancy prevalence remains high compared with other high-income countries,^25^ and there is more to be achieved. Some have suggested, because improvements in use of effective contraception seem to have contributed to the decrease in rates, that the policy emphasis should be placed where it has been successful in the past. Higher contraception discontinuation rates in young people^26^ favour their use of long-acting reversible contraception and additional progress could be made by accelerating the rate at which they are used after onset of sexual activity. Despite the increase in use of these methods, they are used by barely one in six teenage women. The strong and sustained association between conception in women younger than 18 years and earlier sexual activity suggests that sizeable additional gains might also be made by helping young women to become sexually active at a time that is right for them.^27^ A third of all young women, and 60% of those for whom first sex occurs before age 16 years, subsequently consider that to have been too early for them. Bringing actual timing of first sexual encounter in line with preference for timing of first sexual encounter could cut teenage pregnancy rates further, and an intensified policy focus aimed at achieving this might be warranted.

Our data also underline the importance of continued efforts to prevent teenage pregnancy. The decrease in conception rates in women younger than 18 years was more substantial in the later years of the Teenage Pregnancy Strategy and before then, there was scope for detractors to dismiss it as having failed to reach its goals.^28^, ^29^ Our interim study of the assessment of the Teenage Pregnancy Strategy in 2006 showed modest falls in the first 4 years of the strategy, albeit more marked in poorer areas of the country^30^ in which government investment had been greatest. From 2007, however, pregnancy rates began to decrease more steeply and the downward trend continued after the strategy was mainstreamed in 2010. The acceleration is partly explained by the likelihood that strategy-related intervention took time to be implemented. It might also reflect the renewed efforts mounted in 2006 to mobilise additional resources and to persuade senior stakeholders to prioritise the issue in areas in which multiple socioeconomic problems were impeding progress, which is coincidental with a steeper decline in conception rates in women younger than 18 years in these areas in the later years of the Teenage Pregnancy Strategy. A further factor possibly contributing to the decrease in conception rates in women younger than 18 years is the synergy between strategy-related initiatives and other therapeutic and policy-related intervention—the availability of emergency contraception without prescription, for example, the recommendation by the National Institute for Health and Care Excellence that access to long-acting reversible contraception should be increased, and the rise in educational attainment in young people. Taken as a whole, the evidence underlines the importance of long-term, sustained, multifaceted prevention strategies to tackle the more intractable public health challenges.

## Figures and Tables

**Table 1:** Meta-regression analysis of the association between total Teenage Pregnancy Strategy funding per head and absolute and percentage change in the conception rate in women younger than 18 years between 1994–98 and 2009–13

	**Absolute change in conception rate in women <18 years per £100 per girl LIG spend (95% CI)**	**p value**	**Percentage change in conception rate in women <18 years per £100 per girl LIG spend (95% CI)**	**p value**
Unadjusted	−11·4 (−13·2 to −9·6)	<0·0001	−8·6% (−11·9 to −5·4)	<0·0001
Adjusted for region	−11·5 (−13·5 to −9·5)	<0·0001	−8·3% (−11·4 to −5·1)	<0·0001
Adjusted for region and deprivation	−8·2 (−10·5 to −5·8)	<0·0001	−6·2% (−10·2 to −2·3)	0·0023

LIG=Local Implementation Grant.

**Table 2:** Characteristics of women aged 18–24 years in England in Natsal-2 and Natsal-3 and factors associated with conception in women younger than 18 years

		**1999–2001**						**2010–12**					
		Number of participants (unweighted, weighted)	Percentage of participants with characteristic*	Percentage who had conception at age <18 years (95% CI)	OR for <18 years conception			Number of participants (unweighted, weighted)	Percentage of participants with characteristic*	Percentage who had conception at age <18 years (95% CI)	OR for <18 years conception		
					OR	AOR (95% CI)	p value				OR	AOR (95% CI)	p value
Total <18 years conception prevalence		967, 993	..	13% (10·8–15·6)	..	..	..	1368, 817	..	11% (9·1–12·4)	..	..	..
Area-level deprivation							0·008						0·005
	1 (least deprived)	113, 115	12%	3% (1·3–8·5)	1	1	..	219, 131	16%	6% (3·1–9·9)	1	1	..
	2	115, 124	12%	10% (5·3–18·4)	3·23	3·4 (0·98–11·77)	..	227, 133	16%	8% (5·0–11·3)	1·37	1·18 (0·51–2·74)	..
	3	137, 156	16%	8% (4·7–13·7)	2·56	2·2 (0·78–6·22)	..	259, 164	20%	7% (4·4–10·7)	1·25	1·37 (0·55–3·39)	..
	4	229, 256	26%	14% (9·5–19·3)	4·58	3·48 (1·22–9·97)	..	307, 194	24%	13% (9·4–16·8)	2·43	2·15 (0·94–4·89)	..
	5 (most deprived)	373, 342	34%	19% (14·7–24·3)	6·82	5·18 (1·91–14·05)	..	356, 195	24%	17% (13·5–21·8)	3·51	2·89 (1·25–6·67)	..
Quintiles of baseline teenage pregnancy rate							0·387						0·3182
	1 (low)	176, 203	21%	11% (7·1–16·6)	1	1	..	249, 154	19%	8% (5·5–12·4)	1	1	..
	2	190, 188	19%	12% (7·7–18·7)	1·12	0·79 (0·34–1·80)	..	252, 139	17%	14% (10·1–18·4)	1·75	1·16 (0·58–2·31)	..
	3	211, 211	21%	15% (10·6–21·5)	1·47	0·83 (0·38–1·80)	..	242, 134	16%	10% (6·9–15·0)	1·26	0·72 (0·33–1·60)	..
	4	216, 252	25%	14% (8·8–20·7)	1·29	1·04 (0·50–2·18)	..	304, 185	23%	10% (7·4–14·1)	1·26	0·66 (0·32–1·36)	..
	5 (high)	171, 136	14%	11% (6·9–18·1)	1·04	0·52 (0·23–1·18)	..	320, 204	25%	11% (7·8–15·0)	1·35	0·67 (0·31–1·43)	..
Academic qualifications in participants ≥17 years							<0·0001						<0·0001
	Studying/gained further qualifications	435, 475	50%	4% (2·1–5·9)	1	1	..	825, 523	66%	5% (3·4–6·1)	1	1	..
	None, or those typically gained at 16 years†	500, 483	50%	23% (19·1–27·1)	7·99	5·61 (2·97–10·62)	..	510, 266	34%	23% (19·4–27·0)	6·24	3·61 (2·29–5·67)	..
Heterosexual intercourse before age 16 years							<0·0001						<0·0001
	No	693, 713	72%	7% (4·8–9·0)	1	1	..	884, 564	71%	5% (4·1–7·0)	1	1	..
	Yes	270, 274	28%	29% (24·0–35·3)	5·86	3·33 (2·04–5·45)	..	452, 233	29%	24% (20·2–28·6)	5·63	3·2 (2·03–5·04)	..
Reliable contraception used at first sex‡							0·0032						0·1906
	Yes	701, 724	82%	12% (9·4–14·7)	1	1	..	990, 584	87%	11% (9·0–12·9)	1	1	..
	No	163, 156	18%	27% (19·7–35·3)	2·73	2·09 (1·28–3·40)	..	160, 87	13%	22% (16·2–28·8)	2·3	1·44 (0·83–2·48)	..
View on timing of first sex							0·079						0·0693
	About the right time/too late	508, 520	59%	10% (7·4–13·4)	1	1	..	726, 439	65%	9% (7·2–11·4)	1	1	..
	Too early	353, 357	41%	21% (16·9–25·9)	2·39	1·6 (0·95–2·70)	..	426, 232	35%	18% (15·0–22·4)	2·26	1·56 (0·96–2·52)	..
Main reason for first sex							0·1782						0·7324
	Autonomous reason	713, 723	85%	14% (11·8–17·5)	1	1	..	932, 551	83%	11% (9·5–13·6)	1	1	..
	Non-autonomous reason§	128, 130	15%	15% (9·4–22·1)	1·01	0·58 (0·26–1·29)	..	205, 111	17%	17% (12·6–23·6)	1·64	1·09 (0·66–1·81)	..
Partner more willing at first sex							0·356						0·1182
	No	650, 673	77%	13% (10·4–15·8)	1	1	..	970, 564	83%	12% (10·4–14·6)	1	1	..
	Yes	211, 204	23%	20% (14·7–26·7)	1·7	1·27 (0·76–2·13)	..	203, 118	17%	14% (9·5–19·0)	1·12	0·62 (0·33–1·13)	..
Sex education source							0·2297						0·0478
	School lessons	253, 281	29%	9% (5·4–13·7)	1	1	..	519, 314	38%	8% (6·0–10·3)	1	1	..
	Parents	180, 193	20%	13% (8·4–18·5)	1·51	1·73 (0·75–3·99)	..	205, 119	15%	11% (7·3–15·4)	1·4	1·01 (0·48–2·11)	..
	Other	518, 502	51%	16% (12·7–19·7)	1·99	1·69 (0·92–3·10)	..	639, 383	47%	13% (10·3–15·7)	1·71	1·71 (1·06–2·75)	..
Needed more information at first sex							0·1033						0·3937
	No	223, 232	24%	7% (4·1–11·8)	1	1	..	331, 207	28%	8% (5·9–11·6)	1	1	..
	Yes	711, 727	76%	15% (12·7–18·7)	2·43	1·79 (0·89–3·61)	..	930, 535	72%	12% (10·4–14·7)	1·56	1·25 (0·75–2·10)	..
Communication with parents about sex							0·5468						0·9317
	Easy	328, 349	36%	12% (8·4–16·4)	1	1	..	428, 256	32%	10% (7·6–13·4)	1	1	..
	Difficult/not discussed/varied	616, 626	64%	13% (10·3–16·5)	1·12	1·22 (0·64–2·31)	..	896, 543	68%	10% (8·1–12·1)	0·98	0·98 (0·58–1·65)	..
Lived with both natural parents¶							0·2437						0·0088
	Yes	683, 714	72%	11% (8·2–13·4)	1	1	..	826, 527	65%	7% (5·2–8·6)	1	1	..
	No	284, 279	28%	19% (15·0–24·6)	2·04	1·31 (0·83–2·08)	..	541, 289	35%	18% (14·8–21·3)	3·02	1·72 (1·15–2·57)	..

Conception before age 18 years was classified as livebirths occurring before age 18 years 9 months and abortions occurring at age 17 years and younger. Miscarriages were excluded because of common misreporting, in common with Office of National Statistics. Stillbirths excluded for comparability between Natsal-2 and Natsal-3. OR=odds ratio. AOR=adjusted odds ratio.

* Percentages were calculated for weighted participants.

† English General Certificate of Secondary Education or equivalent.

‡ Contraceptive pill or condom.

§ Non-autonomous reason for first sex defined as copying peers or being under the influence of alcohol in Natsal-2 and copying peers or being under the influence of alcohol or recreational drugs in Natsal-3.

¶ Until age 14 years in Natsal-2 and until age 16 years in Natsal-3. AOR is adjusted for whether participants lived with both natural parents until age 14 years or 16 years, ease of communication with parents about sex, Index of Multiple Deprivation, educational attainment, main source of sex education, whether more information was needed at first sex, contraceptive method use at first sex, whether both partners were equally willing at first sex, autonomy of the reason for first sex, and baseline conception rate at younger than 18 years (area-level), but not adjusted for first sex before age 16 years.

**Table 3:** Current participation of women aged 18–24 years in England by experience of early motherhood (1990–91, 1999–2001, and 2010–12)

	**No experience of motherhood resulting from conception before age 18 years**		**Experience of motherhood resulting from conception before age 18 years**		**p value (from χ^2^ test)**
	Denominators (unweighted, weighted numbers)	Percentage participation in education, work, or training (95% CI)	Denominators (unweighted, weighted numbers)	Percentage participation in education, work, or training (95% CI)	
1990–1991	1135, 1393	79% (76·1–81·7)	180, 134	20% (14·1–28·5)	<0·0001
1999–2001	862, 910	82% (79·0–84·8)	106, 81	22% (14·4–33·1)	<0·0001
2010–2012	1203, 738	80% (77·0–82·2)	104, 43	36% (27·1–47·0)	<0·0001
